# What do We Know about Neonatal Cognition?

**DOI:** 10.3390/bs3010154

**Published:** 2013-02-27

**Authors:** Arlette Streri, Maria Dolores de Hevia, Véronique Izard, Aurélie Coubart

**Affiliations:** 1 Paris Descartes University, Sorbonne Cité, Paris, 75006, France; 2 Laboratory for Psychology of Perception, UMR CNRS 8158, Centre Biomédical des Saints-Pères, Paris, 75006, France

**Keywords:** newborn, perception, cognition, memory

## Abstract

Research on neonatal cognition has developed very recently in comparison with the long history of research on child behavior. The last sixty years of research have provided a great amount of evidence for infants’ numerous cognitive abilities. However, only little of this research concerns newborn infants. What do we know about neonatal cognition? Using a variety of paradigms, researchers became able to probe for what newborns know. Amongst these results, we can distinguish several levels of cognitive abilities. First, at the perceptual or sensory level, newborns are able to process information coming from the social world and the physical objects through all their senses. They are able to discriminate between object shapes and between faces; that is, they are able to detect invariants, remember and recognize them. Second, newborns are able to abstract information, to compare different inputs and to match them across different sensory modalities. We will argue that these two levels can be considered high-level cognitive abilities: they constitute the foundations of human cognition. Furthermore, while some perceptual competencies can stem from the fetal period, many of these perceptual and cognitive abilities cannot be a consequence of the environment surrounding the newborn before birth.

## 1. Introduction: Some Historical Markers

Studies of newborn infants have historically been at the heart of a number of controversies (For a more complete history and presentation of different paradigms used in infancy research, see [1]). In Darwin’s Theory of Evolution [2], the characteristics of behaviors present in newborn infants could be ascribed to heredity, and those acquired subsequently were attributable to environmental influences. How could researchers determine and measure what was innate and immutable, and distinguish it from what was acquired and modifiable by training or by the environment? For several decades, the observation of newborns’ and older infants’ motor behaviors was the only visible measure for understanding the development during infancy and childhood. These behavioral responses allowed authors to describe motor and “mental” (“cognitive” at the present) competences of infants and children. The changes of behaviors were ascribed either to maturation [3], or to learning or conditioning [4]. Alternatively, researchers used standardized tests as another means to grasp infants’ and children’s changing minds. For example, the Development Quotient (D.Q.), stemming from Gesell, Thompson and Amatruda’s normative theory [5] of infants’ behavioral development, is measured thanks to Bayley’s test [6]. Infant’s psychomotor achievements were presumed to predict their mental endowment. For school age children, studies of the I.Q. (Intelligence Quotient) were introduced to measure individual cognitive abilities. Because these measures were found to be stable, it was thought to reflect an innate endowment. 

Beyond this heredity-environment dichotomy, and through the observations of his own infants, Piaget [7,8] attempted to understand the emergence and development of the human mind. According to this author, human intelligence comes from the actions “in and on the world” and not from perception, because perception is too ambiguous and poor in young infants. In this theory, perception of light exists from birth, but perception of forms, size, positions, distances, *etc.*, is acquired through the mixing of visual reflex activities such as saccades, fixations and higher activities. In this view, the newborn’s vision is exceptionally impoverished and only after a long period of development and learning, the infant’s vision becomes mature. In contrast, it is the newborn’s sensorimotor reflexes, such as the sucking reflex and grasping, that through practice will become more mature actions, so that an exchange between the newborns and the properties of the objects is established. By acting on the objects, infants are able to abstract information from the properties of the objects and their structure, and to categorize and progressively construct their own representation of the world, during the first two years after birth. Hence, infants’ abilities could not be fully ascribed either to a biological heritage, since infants’ interactions with their environment were crucial in the emergence of these abilities, nor to the environment, since sensorimotor reflexes were necessary to seed the process. Object permanence, which Piaget thought was acquired at about 8–9 months, was considered to be the beginning of a stage where the infant is able to form representations of the world, since he/she is able to memorize the location of a hidden object. Memory and representation are strongly linked in Piaget’s theory. 

It is around the early 60s’ that developmental researchers developed new methods to probe infants’ perceptions (for instance, Fantz’s studies [9,10]). In particular, researchers used infants’ looking times as indices of perceptual activity and of their interactions with the environment. At this point, the field of cognitive development experienced a paradigm shift, as according to Kuhn’s [11] theory. Studies on infants’ perception and cognition became predominant over studies on their sensorimotor abilities. These studies demonstrated that young infants are able to perceive and understand the properties of the environment before they are able to actively act on it. For example, object permanence has been demonstrated in 2 ½ -month-old infants [12]. In fact, and thanks to this change of paradigm, the description of the researchers shifted from a deaf, blind, and confused young infant to an intelligent and competent one [13]. In 1985, Mehler and Fox made this paradigm shift clear by publishing a book entitled “Neonate Cognition: Beyond the blooming, buzzing confusion” [14]. This point marked the beginning of the theory of the initial state, nowadays refuted based on studies of fetuses. However, despite the fact that this theoretical and experimental book emphasized the state of the mind at birth, only a few investigations at this time had been devoted to neonatal cognition. 

## 2. What is Neonatal Cognition?

In a broad sense, ‘cognition’ can be defined as the manner in which the human mind is organized according to its perceptions, memories, beliefs, concepts, learning, knowledge, *etc.* In a narrow sense, “cognition” is sometimes linked to the concept of “representation”, because forming a representation creates a memory, since the information that is actively represented is stored in the brain. In the first infant studies, a crucial question was addressed: Do very young infants perceive the world as human adults do? For human adults, natural scenes reveal a world that is stable, coherent, organized and meaningful, a world of familiar objects, of moving faces and bodies, and of events. How do newborns make sense of the wealth of stable or moving things, events, and people, which they encounter through the senses of audition, vision, touch, and olfaction? 

Plausibly, even though their senses do not have the same acuity as that of adults, newborn infants should possess some means to form a coherent, yet incomplete, representation of these objects, events, and people. In fact, human newborns may be endowed with a large collection of cognitive predispositions that allow them to extract and represent invariants in their environment. In particular, newborns may be prepared to interpret their sensible experience with representations of conceptual content (see [15]). We assume that some concepts are innate but not inevitably genetically determined. 

Bremner [16] claimed that “… the environment has a permitting role in development, but it doesn’t have a determining role”, when referring to infant cognition. To qualify this proposition concerning the behavioral studies of neonatal cognition, we will call *innate* all the behaviors that are revealed by the environment, while we will refer to all behaviors that depend or are instructed by the environment as *learned*. For example, an infant may be able to see as soon as light reaches his/her retina; but to see shapes or objects, he/she will need to process the structured information of light reflected by these objects. If, a few minutes after birth, newborns are able to see a structured target (a shape or object), then it means that the newborns’ eyes and cortical brain are prepared to see this target. This competence is *revealed* by the environment. In contrast, if the newborns’ vision needs multiple inputs during several weeks to see the structured target, then this competence is learned and *instructed* by the environment. 

Even though they are present at birth, for them to be innate they should not be the result of learning during the fetal period. Below, we develop some examples of such predispositions. We will review evidence that newborn infants are able to represent and store information extracted from the outside world through their sensory modalities, and to give meaning to the events that they perceive. For instance, newborns are prepared to see objects in a 3D environment; they can extract amodal information such as numerosity; more generally, the information extracted from the visual modality is predisposed to be linked to other modalities immediately after birth. Since infants are able to learn very quickly, in the present review we mainly focus on two to three day-old neonates. 

## 3. Neonatal Perception

Perception is the gateway through which energy and matter in the physical world lead to ideas in the mind. All the senses emerge and function in humans early during gestation, except for visual receptors that mainly function at birth, possibly due to the relatively low level of stimulation the eyes receive in utero. Perception starts as the sensory systems enter in contact with the environment, be it fetal or neonatal. 

Thanks to the habituation/dishabituation paradigm, it is feasible to demonstrate that all senses are functional from birth. In habituation studies, mainly administered in the visual modality, the duration of fixation to a particular display is measured over several trials. Habituation refers to the fact that the infants’ visual attention to a particular stimulus declines over time. If a different stimulus is presented, recovery of looking time—dishabituation- will occur. Habituation is a simple form of learning, and it refers to the decline of attention to a repeatedly presented target. It means that the infant is able to remember what he/she sees [17]. Furthermore, in order for dishabituation to take place, infants need not only to detect what is invariant across the repeating trials (in the course of habituation), but they must also detect that something has changed during the test trials. It is now well established that, because habituation involves a memory and a comparison of stimuli, this paradigm can demonstrate the existence of representations in the mind of infants (cf. [18,19]). 

The habituation/dishabituation method allowed researchers to address several important questions about infants’ perception. In fact, all senses can be habituated and dishabituated in the same manner. For example, in the visual modality the discrimination of orientation was evidenced at birth [20]: In this study, newborns were habituated to a diagonal grating (tilted either clockwise or anticlockwise from the vertical), and subsequently they were observed to react to the novelty present in a mirror-image of the previously seen grating. Moreover, numerous studies have provided evidence for the presence of a structured visual perception in newborns. For example, nine minutes after birth, newborns are able to perceive patterns of faces [21], discriminate between curvilinear and rectilinear 2D forms (see [22] for a review), conceive the unity of partially hidden objects [23], or discriminate small, (2 *vs.* 3 black dots) but not large, (4 *vs.* 6) numerosities [24]. Concerning the “sense of numerosity”, this competence is highly shared by other non-human animals, even in newborns. For example, one-day-old fish can discriminate between quantities of social companions, even in absence of previous social experience [25]. 

The preferential looking paradigm, introduced by Fantz [8], is another method to evaluate the newborn’s competencies, although it can be used only in the visual modality and not in the other sensory modalities. This method tests for the discrimination and “preference” between two pictures, faces, or objects from birth. For example, when presented with face and non-face configurations, newborns spontaneously look longer at face-like patterns [26,27,28,29]. More precisely, newborns are sensitive to the presence of small cues in the faces, such as gaze orientation and mouth configuration. Newborns look longer to a face that displays a direct gaze than to one that displays an averted gaze. This preference is exhibited both for static faces [30,31], and for adult faces that speak to them [32]. By manipulating the contrast polarity of schematic and naturalistic faces, Farroni and colleagues [33] revealed that newborns show a preference for upright faces, unless they contain darker areas around the eyes and the mouth. 

While the visual preferential looking paradigm does not involve memory, as in the habituation paradigm, a combination of the two methods is possible, particularly in bimodal auditory-visual situations. In a recent study, newborns were presented during the habituation phase with a speaking face presented on a video screen for 90 seconds [34]. In the test phase, newborns saw a photograph of the familiar face and of a novel face. Newborns looked longer at the familiar than at the novel face. In a second experiment, the sound was removed during familiarization and newborns saw instead a woman who moved her lips without emitting speech sounds. Under these conditions, there were no differences in looking times between novel and familiar faces during the test phase, thus revealing that newborns did not recognize the familiar face. Speech appears to be crucial for newborns in order to recognize a face.

The habituation paradigm has also been exploited in order to test newborns’ abilities in the tactile modality. Full term newborns, and even preterm newborns, are able to hold an object in their right hand without visual control and sometimes to palpate it. A decline of holding time is observed when the object has been repeatedly presented, which signals that infants have habituated to that stimulus. The presentation of a novel object to the newborn’s hand can reveal a dishabituation phenomenon (reaction to novelty). These habituation and dishabituation processes for the tactile modality have been observed with both the left and the right hands [35], and have been described also in preterm infants [36].

Investigating newborns’ competencies in the auditory modality is crucial, if we consider that the ability to discriminate sounds is a prerequisite for normal speech development. For decades, behavioral methods, such as the head-turning and the sucking paradigms, have been the primary methods used to investigate auditory discrimination, learning, and the function of sensory memory in newborns and young infants (see [37,38,39]). In fetuses, the responses to a sound are measured by the recording of acceleration and deceleration of their cardiac rhythm. In all the cases, the indexes of infant’s auditory responses are indirect in contrast to the visual or tactile modalities, in which the sensory system directly responds to the stimulation. 

Audition is excellent at birth, because it is already functional in utero. By 35 weeks of gestation, cochlear biomechanics and frequency selectivity are already mature (see [40]). Near-term fetuses can discriminate between two pure tones [41], and two low-pitched piano notes [42]. Concerning the perception of complex sounds like speech and music, fetuses can discriminate differences in Sound Pressure Level (SPL), frequencies, and spectra, which are necessary for perceiving melodic contours and prosodic features of speech. For example, they can discriminate between a male and a female voice contrasted on fundamental frequency, spectra, and timbre [43]. Because speech sounds are well heard in utero, the auditory memory is excellent at birth. For example, when presented with their mother’s voice and with a stranger’s voice, newborns exhibit a strong preference for their mother’s voice [44]. They also prefer listening to their native language when contrasted to others [45,46]. Familiarization (habituation) and recovery to sounds have been assessed by spontaneous head orienting towards the sound’s location. With this method, Swain and colleagues [47] have shown that newborns that have heard a specific sound during one hour appear to retain it over a 24-hour period when presented with the same sound the next day. 

During recent years, new methods have been used for investigating more directly neurobiological bases of speech discrimination. These methods make use of the mismatch negativity (MMN) revealed for the first time by Näätänen and colleagues [48]. The investigation of sound discrimination and related cortical activity of the fetuses and newborn infants can help to identify and determine the nature of deficits caused by central processes in the auditory system at very early stages. The MMN, a preattentive, change-specific component of the auditory event-related potential (ERP), is elicited when a physically deviant sound is infrequently presented among the repetitive (standard) sounds (see for a complete review [49]. Event-related potentials are often recorded from the scalps of sleeping, and sometimes awake newborns. Magnetoencephalography (MEG) is also suitable for neonatal assessment of auditory discrimination (see [50]). The MMN presents some similarities with the habituation/reaction to novelty method used in behavioral studies, except that the habituation/reaction to novelty method is only possible when infants are awake. 

A good amount of evidence has revealed that newborns are able to identify repeated auditory feature conjunctions even when such conjunctions occur rarely among other similar ones [51], as well as to detect pitch changes in a repetitive sequence of tone pips [52]. Using MEG, fetuses and newborns have been shown to be sensitive to a difference between a standard complex tone of 500 Hz intermixed with a deviant complex tone of 750 Hz, as well as to four types of acoustic changes, such as frequency, intensity, duration, and a gap presented within a sound stream [53]. Using NIRS, Pena and colleagues [54] provided evidence for left hemisphere dominance for speech in the newborn’s brain. Newborns discriminate the vowel /o/, presented as standard, from the vowel /e/, which was the deviant, both during sleep and wakefulness [55]. Moreover, newborns discriminate a frequent vowel sound [a:] from an occasionally presented vowel [i:], thus supporting the results observed by Coulon, Hemimou and Streri [56] with the neonatal imitation paradigm. Finally, newborns are able to detect fine differences in durational changes for speech sounds, for example, fricative /s/ within bisyllabic nonsense words (/asa/ *vs.* /assa/) [57]. Auditory memory has also been revealed by MMN in awake or asleep newborns, but the time span of auditory memory is considerably short in newborns [58]. This result contrasts with that observed by Swain and colleagues [47], *i.e.*, a 24-hour period of retention of sounds, plausibly because these sounds were repeated during a long time in the habituation phase. 

Whichever the different techniques or procedures used, either behavioral (habituation, visual preference) or brain imagery (ERP, MEG), newborns have been shown to be able to process inputs immediately perceptible by their senses. However, all the information coming from the environment is not directly perceived by our senses. For example, we do not see all the aspects of a 3D object or all sensory aspects (tactile, auditory, *etc.*) of a seen object. In fact, some attributes are missing, and therefore it is necessary to complete this information in order to obtain a clear representation of the objects and events. Are newborns able to do this process? In what follows, we present two case examples of this process: the perception of 3D space, and the integration of information coming from different sensory modalities. Understanding these processes can shed light on the capacities that newborns possess.

## 4. Neonatal Cognition: Perception of 3D Space

Spatial perception is one of the more fundamental cognitive abilities that human beings possess. If objects and events were not organized in a three-dimensional framework, the environment would not appear coherent, but rather confused and chaotic, dependent on the sensory receptors. Therefore, an innate predisposition to project percepts in a 3D space would be especially helpful for infants to make sense of their sensory input. Beyond these sensory abilities mentioned above, Slater, Mattock, and Brown [59], as well as Granrud [60], provided convincing evidence showing that newborn human infants are sensitive to the true size of objects, despite changes in retinal size (size consistency). In order to recognize size constancy, infants need to combine projective size (visual angle) with information about viewing distance [61]. In two experiments testing size constancy in newborns, Slater and colleagues [59] familiarized two groups of newborns to one cube (either a large or a small cube). During this phase, the object was presented at different distances from the eyes. Then, in the test phase, both cubes (large and small) were presented to the two groups simultaneously, but at different viewing distances, such that the sizes of their projection on the retina were identical. Infants of both groups looked more at the cube that differed in size from the familiarized cube (either the small or the large cube depending on the familiarization condition), despite the fact that the projected sizes were identical. 

This example also illustrates the need for inference in perception. In the present experiment, newborns could see the difference in the objects while retinal stimulation remained unchanged. Empiricist and constructivist positions have held that visual stimulation is inadequate to produce depth perception, and that reliance on other information, such as tactual sensations or actions, is required to interpret vision. In contrast, newborns are sensitive to depth and can perceive an object in a three-dimensional space immediately after birth, in the absence of previous experience exploring the world in the tactile modality. It is a remarkable evidence of a competence revealed by the environment. 

Research on auditory space perception also suggests that a partial ability to spatially localize objects based on a source of sound is present at birth. Newborns have been shown to know the direction in which a sounding object is located [62]. However, perceiving the direction of a sound source does not allow knowing its location in a three dimensional space, because the cues that specify the direction do not contain information about the distance. Reaching a sounding object in the dark is a better index of perception of auditory space and orientation, but this performance appears to emerge later, at around 6 months of age [63]. 

In the visual modality, while there is evidence that newborns are endowed with mechanisms that allow them to see the world in a three-dimensional frame, the stereoscopic depth perception seems to appear later in infancy, at around the fourth month of life [64], and arises after the same amount of visual experience in full-term newborns and in preterm infants [65]. The most likely mechanism determining the emergence of stereoscopic vision is more central, and possibly due to some maturational changes in cortical disparity-sensitive units. 

Although stereoscopic depth space does not emerge directly at birth, research has revealed that newborns are able to spontaneously (without familiarization) detect similarities and differences between three-dimensional stimuli and their two-dimensional representations [66]. In either the visual preference or the habituation procedures, newborns look more at complex objects than at their photographs, both in binocular and monocular vision. It is plausible that motion parallax is a salient cue having a role in the detection of these differences. 

In summary, research on young infants’ perceptual abilities has revealed that from birth on, infants are able to (1) form a memory and learn about their environment—as evidenced in habituation/dishabituation paradigms, and (2) extract stable invariants from a three-dimensional world, and have the intuition of space and of the unity of objects.

## 5. Neonatal Cognition: Multimodal Sensory Integration

Because the world is multimodal, information arising through the different senses needs to be merged in order to form representations of the objects that contain all their properties, tactile, auditory, visual, *etc.* Contrary to the empiricist hypothesis, which states that infants need to progressively learn to combine various sensory inputs, Gibson, E.J. [67] proposed that spatial dimensions, as well as temporal dimensions, are amodal in nature, *i.e.*, they are available to all sensory modalities right from birth. Amodal perception occurs whenever two or more senses provide equivalent information. In fact, it is quite likely that the ability to detect amodal relations is innately given to the infant. For instance, Wertheimer [62] reported consistent eye movements in the direction of an auditory stimulus positioned close to either the left or right ear of a 10-min-old infant. He interpreted these ipsilateral eye movements as providing evidence for an innate mechanism subserving the integration of visual and auditory information. 

In some cases, newborns’ preferential orientation towards a stimulus can stem from prenatal life. Chemosensory interactions provide a good illustration of multisensory interactions because several senses are involved during the fetal period. In newborns, volatile components of flavor, detected by the olfactory system, are strongly influenced by early exposure in utero. Chemical molecules soluble in the amniotic fluid soak in continuously through the nose, lips and tongue of the fetus. The fetus can detect and store the unique chemosensory information available in the prenatal environment. In fact, at birth, when exposed to paired-choice tests contrasting the odors of familiar or non familiar amniotic fluids, infants orient preferentially and selectively to the odor of familiar amniotic fluid [68]. Thus, from the volatile, and non-soluble, fragrant information alone, newborns are able to recognize the composite chemical fluid information they have learned in utero. 

The question of whether there are links between sensory modalities in newborns is crucial because although both haptic and auditory information is available during the fetal period, visual information is not. Furthermore, when links between sensory modalities are present at birth, these interactions can be ascribed to different levels, from sensory to cognitive, revealing the existence of abilities that newborns possess, and that allow them to understand the environment. For example, at a low level, auditory stimulation can modify and influence the infant’s state of arousal and preference for visual stimuli. Lewkowicz and Turkewitz [69] have demonstrated that newborns that were first exposed to light spots of different intensities, looked preferentially towards a light of intermediate intensity. In contrast, newborns that were first exposed to a sound (white noise), and then to various light spots, seemed to prefer the light with the lowest intensity. Lewkowicz and Turkewitz [69] concluded from these results that newborns attend to quantitative variations in stimulation. Moreover, they suggested that newborns ignore the qualitative attributes of the stimulation in favor of the quantitative ones. 

At a different level, newborns are able to perceive the equivalent nature of the visible and audible aspects of an event and to integrate the multimodal properties of temporal events into unified experiences. From birth, temporal synchrony appears to be a fundamental cue for the perception of an inter-sensorial unit [70]. Many multisensory events provide both amodal and arbitrary auditory-visual information. Infants’ learning of these auditory-visual intersensory relations, both amodal and arbitrary, has been investigated in detail in the studies by Bahrick [71,72,73]. Morrongiello and colleagues [74] have demonstrated that newborns can associate objects and sounds based on the combined cues of collocation and synchrony. They are even able to learn arbitrary auditory-visual associations (e.g., between an oriented colored line and a syllable), but only in the condition where the visual and auditory information were presented synchronously [75]. All these results suggest that by means of the temporal and spatial synchrony or co-occurrences available in the visual and auditory information, newborns possess the foundations of the perceptual mechanisms that will allow them, later, to learn the meaning of lexical words. 

Finally, while temporal synchrony is a fundamental dimension in order to establish the link between visual and auditory information about an event or an object, this is not always the case when amodal information is presented to newborns. For example, Izard and colleagues [76] recently revealed that newborn infants spontaneously associate slowly moving visual-spatial arrays of 4–18 objects with rapid auditory sequences of events based on number. In these experiments, infants were familiarized with sequences of either 4 or 12 sounds (6 or 18 sounds) accompanied by visual arrays of either 4 or 12 objects (6 or 18 objects). These images were animated with a regular saccadic movement, which was not synchronic with the presentation of the sounds. For all the familiarization conditions, however, newborn infants looked longer at the visual image containing the matching number of objects. Despite the absence of synchrony between the sounds and the objects, newborns were able to respond to abstract numerical quantities presented across different modalities (auditory and visual), and formats (*i.e*., sequential *vs.* simultaneous), suggesting that newborns were not treating this relation as arbitrary but as consisting of the same information, even though coming from different modalities. 

In the same manner, we may ask whether human newborns can perceive the equivalence of shapes across the visual and tactile modalities. For several centuries, the question of crossmodal integration between the senses has been addressed by philosophical answers to Molyneux’s famous question: Will a person born blind who recovers sight as an adult immediately be able to visually distinguish a cube from a sphere [77,78]? Molyneux’s question precisely describes the crossmodal transfer task from the hands to the eyes in infancy. Although newborns are not directly comparable to congenitally blind individuals, if they are able to form a perceptual representation of the shape of objects from the hand, and to recognize this shape visually, this would suggest an affirmative answer to Molyneux’s question. A successful transfer would mean that a link between the hands and the eyes exists before infants have had the opportunity to learn from the pairings of visual and tactual experiences.

The crossmodal transfer tasks used with newborn infants involve two successive phases: a habituation phase in one modality, followed by a recognition phase in a second modality. In the “tactile-to-visual modality” task, newborns undergo tactile habituation to an object, which is placed in their hand. Then, in the second phase, the familiar and the novel objects are visually presented in alternation during four trials in a counterbalanced order between participants. This paradigm, widely used in infancy studies, involves several cognitive resources. In the first phase, the baby has to collect a piece of information on an object in one modality, memorize this information, and then, in the second phase, recognize which object is the familiar one in a different modality.

Streri and Gentaz [79,80,81] conducted an experiment on crossmodal recognition of shape from the hand to the eyes in human newborns. Tactile objects consisted on a small cylinder (10 mm in diameter) and a small prism (10 mm triangle base). Because the vision of newborns is immature and their visual acuity is weak, visual objects were the same, much larger 3D shapes. The results revealed that newborns looked longer at the novel object than the familiar one. These results suggest that newborns recognized the familiar object through a visual comparison process, as well as a comparison between the haptic and the visual modalities. Moreover, the discrepancy between the size of visual and tactile objects was apparently irrelevant for crossmodal recognition: Only shape seemed to be considered by newborns. In conclusion, newborns are able to transfer shape information from touch to vision before they have had the opportunity to learn the pairing between the visual and the tactile experiences. These results challenge the empiricist philosophical view, as well as modern connectionist models [82,83] that argue that sensory modalities cannot communicate between them in newborns. The results reveal an early-developing ability, largely independent of experience, to detect abstract, amodal higher-order properties of objects. This ability may be a necessary prerequisite for the development of knowledge in infancy. Based on these findings, we propose that various sources of perceptual information about objects in the environment are successfully unified at birth and that they enable newborns to perceive the world as stable.

In short, when newborns are presented with bimodal situations (auditory/visual or tactile/visual) in which spatiotemporal synchrony cannot help them to integrate the information to form a coherent event, they attempt to understand the situation and react to it in an appropriate manner. How is this possible? Plausibly, while the cerebral bases of newborn’s cognition might explain these results, no EEG or MEG data are currently available. We suggest that a newborn’s mind is abstract. Newborns are able to abstract the pieces of information common to two modalities, such as shape, in the intermodal transfer from touch to vision, and the quantity in the auditory-visual situation. At the same time, some details are neglected, such as the discrepancy between the size of tactile and visual objects, and the format of presentation of quantities (auditory syllables *vs.* visual smileys). This process allows newborns to immediately perceive the invariants from the new environment to which they must adapt. Newborns think in an abstract manner. 

## 6. Conclusions: Human Newborns are Prepared to Perceive, Memorize and Understand a Complex Environment

Several decades of research have revealed newborns’ cognitive potentialities, providing a picture of a human newborn different from a human immersed in a disorganized world. Nowadays, an increasing number of studies have provided evidence of three different abilities that allow newborns to contact with, and learn something from, their environment: their clumsy actions or scheme-reflexes (in the sense of Piaget’s theory), their perceptions, and lastly, their cognitive abilities. Why did it take so long to better understand the two last mentioned abilities?

Putting aside the question of missing methodological tools, a first theoretical explanation to this problem rests on the assumption that the development of knowledge is absent or relatively simple in newborns, and that it becomes increasingly complex in adults thanks to learning, and culture. This idea is indeed not contradicted by the recent data from newborns. Neonatal perception and cognition are the simplest foundations that will allow infants and children to eventually develop more mature knowledge through the acquisition of motor skills, language, school learning, *etc.* In the course of development, infants and children process information more quickly and more precisely as a function of the maturation of the sensory systems, and the brain structures. The developmental course appears sometimes continuous, and sometimes discontinuous, but it remains unchanged across the lifespan. Examples of continuous development can be taken from the cognitive domain. During infancy, and throughout life, the perception of large numerosities is approximate and governed by Weber’s law. Whereas newborns require a broader ratio (1:3: [76]), 6-month-old infants need a 1:2 ratio to successfully discriminate numerosities, and 10-month-old infants can discriminate numerosities at a finer ratio (2:3: [84]). The object permanence observed at 2 ½ months of age [12] shows that the infant can create the representation of a hidden object. However, this representation, which vanishes if the object is hidden during a long time, will become stronger and more precise (*i.e*., containing the properties of the object) in the course of development. An example of discontinuous development concerns the crossmodal transfer from touch to vision that is present at birth. This ability disappears at 5 months [85], and reappears at 6 months of age. This apparent weakness is due to the emergence of a new ability: the coordination between prehension and vision (for a review of this developmental process, see [86]). 

Finally, because the idea that newborns could see their environment was counterintuitive, as soon as methodological tools allowed it, the researchers simplified considerably the stimuli that were proposed to infants. The first materials proposed to the newborns in experimental studies were black and white, stable 2D stimuli, and each sense, audition, vision, and touch, was studied separately. These studies revealed the early ability to perceive and discriminate information in the environment. However, we would like to argue that the mind of a newborn might be better prepared to extract invariants from complex, multimodal situations. Since newborns must enter in contact with a complex, multidimensional world, they ought to possess some mechanisms that allow them to immediately adapt to that environment. Even though at birth these mechanisms are still weak, clumsy, and primitive, it is thanks to them that newborns develop and adapt directly to their environment. This idea is illustrated at the neural level in Berkes *et al.*’s [87] study on the development of cortical activity of awake ferrets. As the young animals accumulate experience with natural scenes, the spontaneous and evoked neural activity converges towards a common pattern: the animal develops an internal model of the environment progressively, from an initial imperfect model. However, this adaptation is specific to natural scenes, and does not occur when the animal is viewing artificial stimuli such as gratings. These findings illustrate how, although animals need to refine their internal model through learning, they can only do so for the stimuli they are prepared to see. Irrespective of their species, newborns are prepared to adapt to their environment and have the tools to understand it. As for the human newborns, the studies concerning their cognitive abilities are recent and far from having established the full repertoire of competencies that they possess. 
